# Subjective cognitive impairment, cognitive disorders and self-perceived health: The importance of the informant

**DOI:** 10.1590/1980-57642018dn13-030011

**Published:** 2019

**Authors:** Mariana Luciano de Almeida, Daniela Dalpubel, Estela Barbosa Ribeiro, Eduardo Schneider Bueno de Oliveira, Juliana Hotta Ansai, Francisco Assis Carvalho Vale

**Affiliations:** 1 Universidade de São Paulo College of Nursing of Ribeirão Preto Ribeirão PretoSP Brazil MSc, College of Nursing of Ribeirão Preto - Universidade de São Paulo, Ribeirão Preto, SP, Brazil.; 2 Universidade de São Paulo Medicine School of Ribeirão Preto Ribeirão PretoSP Brazil MSc, Medicine School of Ribeirão Preto - Universidade de São Paulo, Ribeirão Preto, SP, Brazil.; 3 Federal University of São Carlos Nursing Department São CarlosSP Brazil MSc, Nursing Department - Federal University of São Carlos, São Carlos, SP, Brazil.; 4 Universidade de São Paulo Federal University of São Carlos Statistic Department São CarlosSP Brazil MSc, Statistic Department - Federal University of São Carlos/Universidade de São Paulo, São Carlos, SP, Brazil.; 5 Federal University of Mato Grosso do Sul Campo GrandeMS Brazil PhD, Federal University of Mato Grosso do Sul. Campo Grande, MS, Brazil.; 6 Federal University of São Carlos Medicine Department São CarlosSP Brazil PhD, Medicine Department - Federal University of São Carlos, São Carlos, SP, Brazil.

**Keywords:** memory complaint, cognitive impairment, older adult, caregiver, queixa de memória, comprometimento cognitivo, idoso, cuidador

## Abstract

There is great divergence of results in the literature regarding the clinical relevance and etiology of subjective cognitive impairment (SCI). Currently, SCI is studied as a pre-clinical symptom of Alzheimer's disease, before establishing a possible diagnosis of mild cognitive impairment (MCI). The hypothesis was that SCI is associated with low cognitive performance and poor self-perceived health.

**Objective:**

to investigate the relationship of SCI with objective cognitive impairment and self-perceived health in older individuals and to compare SCI reported by the elderly subjects and by their respective informants.

**Methods:**

83 subjects participated in the study, divided between the forms of the Memory Complaint Scale (MCS). Cognition was evaluated by the Addenbrooke's Cognitive Examination - Revised and self-perceived health by the Short Form Health Survey-8.

**Results:**

there was no association between SCI and self-perceived health. SCI reported by the older adults was associated with executive functions. SCI reported by the informant was associated with overall cognitive performance, memory, verbal fluency and visuospatial functions.

**Conclusion:**

we found more robust results between SCI reported by the informant and cognitive impairment in the elderly assessed. There is a need to include and value the perception of someone who knows the older individual well enough to evaluate SCI globally.

## RESUMO

Há grande divergência de resultados na literatura em relação à relevância clínica e à etiologia do comprometimento cognitivo subjetivo (CCS). Atualmente, o CCS é estudado como um sintoma pré-clínico da doença de Alzheimer, antes de se estabelecer um possível diagnóstico de CCL. A hipótese é que o CCS pode estar associado a um desempenho inferior em testes cognitivos ou a uma autopercepção de saúde ruim.

**Objetivo:**

O objetivo do estudo foi investigar a relação do CCS com o comprometimento cognitivo objetivo e a autopercepção de saúde em idosos e comparar o CCS relatado pelos idosos e o CCS relatado pelo informante.

**Métodos:**

83 sujeitos participaram do estudo, divididos entre as formas da Escala de Queixa de Memória para as análises. A cognição foi avaliada pelo Exame Cognitivo de Addenbrooke - Revisado e autopercepção da saúde pelo Short Form Health Survey-8.

**Resultados:**

não houve associação entre CCS e autopercepção de saúde. O CCS relatado pelos idosos foi associado com funções executivas. O CCS reportado pelo informante esteve associado ao desempenho cognitivo global, memória, fluência verbal e função visual espacial.

**Conclusão:**

neste estudo encontramos resultados mais robustos entre CCS relatado pelo informante e o comprometimento cognitivo objetivo dos idosos, evidenciando a necessidade de incluir e valorizar a percepção de um indivíduo que conheça o idoso bem o suficiente para avaliar o CCS de maneira integrada.

With the increase in life expectancy, there is a concern about establishing how to discern which factors are natural to the aging process and which are pathological. One of the most distressing factors is cognitive decline. A reflection of this self-evaluation and perception is memory complaints or subjective cognitive impairment.^1^

The prevalence of SCI in community-dwelling older adults ranges from 25 to 50%.^2^ For a better understanding of SCI, the use of a definition of complaints is recommended, using validated and culturally adapted instruments together with evaluation of the elderly subject with the complaint and an informant.^3^

The SCI term is associated with the concept of metamemory, which is the knowledge and awareness that each person holds about how their own memory is generally working. According to the concept, the performance of memory tasks in older adults may also be negatively influenced by their attitudes and beliefs about their ability to memorize.^4^ Although the SCI term is used to refer to a report of memory problems, which may or may not be perceived by others, there is no consensus on a definition for the concept.^5^^,^^6^

Results of studies regarding clinical relevance and etiology of SCI remain conflicting. This disparity is probably due to differences in methodologies among studies in terms of scales used for assessing SCI, prospective or retrospective memory and different populations and contexts. Some studies associate SCI with objective cognitive impairment, suggesting the need for more research, since cognitive impairment may indicate cognitive disorders.^7^^-^11

In addition to the relationship between SCI and objective cognitive impairment, there is a concept that has been studied and indicated as a powerful predictor of SCI, self-perceived health. A Brazilian study^12^ found that people that perceive their health status as poor are more likely to be concerned about their problems. These concerns can lead to attention and concentration disorders, resulting in further memory failure and complaints. SCI is a component of overall health assessments, along with perceived lack of energy, emotional reaction, moodiness, sleep disturbances, pain, social isolation, and problems with physical mobility. Older adults who perceive negative alterations in their overall health are prone to having complaints.^12^

There are still gaps regarding the study of SCI. In this context, the main objective of the present study was to investigate the relationship of SCI with objective cognitive impairment and self-perceived health in community-dwelling older adults. Also, the study sought to compare the SCI reported by the elderly assessed and by their respective informant, according to a new SCI evaluation instrument. The hypothesis of this study is that SCI may be associated with lower cognitive performance and poor self-perceived health.

## METHODS

### Study design and ethical procedures

A cross-sectional, correlational, quantitative study was conducted. The study and the Free and Informed Consent Form were approved by the Ethics Committee for Research in Human of the Federal University of São Carlos (CAAE:34297414.5.0000.5504). All ethical aspects (Resolution 466/12 MS regulated by the National Health Council) were appropriately observed and respected.

### Subjects

This was a simple random sample, extracted from the database of an epidemiological study carried out in a Brazilian city in 2012. For the final sampling procedure of the epidemiological study, a random stratified proportional sample was formed of individuals aged 50 years and older from the city of study. The strata and their quantity were defined by the number of combinations of gender categories and age groups, from the pre-defined age, drawing on the subdivision of the Brazilian Institute of Geography and Statistics (IBGE, 2010) as a reference.

Inclusion criteria were: age ≥65 years and existence of an informant who knew the subject sufficiently well to provide information on questionnaires used to evaluate the individual. Exclusion criteria: individuals with a diagnosis of dementia, mental illness or disorder, untreated systemic diseases that would make participation impossible, uncorrected auditory or visual deficits that would make cognitive tests impossible, individuals scoring >5 on the Geriatric Depression Scale, and those with below-expected cognitive scores for their age and education, evaluated by a specialist neurologist in the area of cognitive disorders. Subjects with a possible MCI diagnoses were included.

The data collection period spanned from January to May 2015, and interviews were conducted at the households of the participants, individually by three different interviewers. All interviewers were duly trained to conduct the interviews and had full knowledge of the protocol applied.

### Data collection instruments

An instrument was used for sociodemographic and clinical characterization of the participants, including sociodemographic and clinical data, such as age, sex, occupation, marital status, education, diagnosed diseases, medications in use, etc. The Brazilian Economic Classification Criteria (CCEB)^13^ were also used to classify the sample into economic classes.

SCI was evaluated using the Memory Complaint Scale (MCS), an instrument composed of seven graded questions of increasing intensity (0, 1, and 2). The total score ranges from 0 to 14 points, minimum and maximum, respectively. The scale is divided into levels of memory complaint, which vary according to the score obtained: No MC (0-2); Mild MC (3-5); Moderate MC (7-10); and Severe MC (11-14). The scale has two versions, one to be applied to the evaluated subject (MCS-A), and the other to their companion or caregiver (MCS-B).^6^

For cognitive evaluation, the Addenbrooke's Cognitive Examination - Revised (ACE-R)^14^^,^^15^ was applied. This is an instrument with high sensitivity and specificity for detecting mild stage dementia, composed of five domains, each with a specific score: orientation and attention, memory, verbal fluency, language, and visuospatial ability. The ACE-R score ranges from 0 to 100 points, where the higher the total score, the better the cognitive status. The ACE-R incorporates the Mini-Mental State Examination (MMSE)^16^ and the Clock Drawing Test (CDT),^17^ which were also used in this study.

Self-perceived health was measured using the Medical Outcomes Study 8-item Short-Form Health Survey (SF-8), which is a reduced version of the SF-36^18^ instrument. The SF-8 includes eight items that address: general health, physical functioning, role physical, bodily pain, vitality, social functioning, mental health, and role emotional. As the SF-8 was prepared similarly to the SF-36, the results of the two assessment instruments can be compared and interpreted using the same interpretation guidelines. The SF-8 total score ranges from 0 to 100 points, where the higher the score, the better the self-perceived health.^19^

The Geriatric Depression Scale (GDS), an instrument for screening depressive symptoms in older adults, was used to avoid possible biases in the influence of depressive symptoms. The short version, with 15 items, was used in this study.^20^

### Statistical analysis

The sample calculation was performed using the G*Power software^®^:^21^ effect size=0.4, a=0.05, b=0.8 and a sample size of n=100 was obtained.

The data obtained were coded and organized into a database with double entry in the Microsoft Excel^®^ program. All analyzes were performed using the free software R.^22^ Descriptive analyses were performed and Spearman's ρ (rho) was used as a correlation coefficient, with formal tests of significance. To perform the formal tests in the contingency tables to verify whether there was significant influence of each categorical variable with respect to the MCS-A and subsequently the MCS-B, the likelihood ratio test was used.^23^^,^^24^ In addition, the analysis in these tables was performed by column profile, allowing better visualization of the effects on each variable regarding the memory complaint.

For the quantitative variables, the non-parametric Wilcoxon Mann-Whitney test was used to verify whether the variables were influenced by the fact that the individual had a memory complaint or not.^25^

To obtain the *Odds Ratio* (OR), together with a new significance test of the covariates, “univariate” logistic regression was applied, i.e., considering the adjustment of models with one covariant at a time as an explanatory variable of the MCS- A and subsequently the MCS-B.

A significance level of 5% was adopted.

## RESULTS

In total, 100 subjects participated in the present study; all with medical records reviewed by the neurologist. From this total, five individuals were excluded due to cognitive performance similar to individuals diagnosed with dementia and 12 subjects rejected for having a GDS score above five points. The final sample comprised 83 subjects, divided into two groups: older adults with SCI (SCI Group) and older adults who did not present SCI (NSCI Group).

The subjects were divided into those with and without memory complaints according to the two MCS forms. Thus, the results related to the clinical variables were divided into two items: MCS-A and MCS-B.

The economic class was analyzed without dividing the subjects as the distribution of this status proved similar for the two groups. The most prevalent economic classes were C1 (30%, n=30), B2 (28%, n=28), and D (14%, n=14), followed by C2 (12%, n=12), B1 (10%, n=10), A2 (5%, n=5), and E (1%, n=1). The other sociodemographic variables were analyzed separately (Table 1).

**Table 1:** Sociodemographic and clinical variables based on SCI levels, according to the MCS-A and MCS-B.

	MCS-A				MCS-B		
	SCI (n=34)	NSCI (n=49)	p		SCI (n=28)	NSCI (n=55)	p
Sex (F)	25	31	0.323^a^		19	37	0.957^a^
Age (mean ±)	73.8 (7.2)	74.2 (7.3)	0.841^b^		75.0 (7.2)	73.5 (7.3)	0.285^b^
Education (mean ±)	5.4 (4.0)	5.3 (4.9)	0.617^b^		5.1 (5.5)	5.5 (4.1)	0.317^b^
Marital status (n)	34	49	0.738^a^		28	55	0.121^a^
Married	24	30			22	35	
Divorced	2	4			1	5	
Widowed	6	13			5	14	
Single	2	2			0	4	

MCS-A: Memory Complaint Scale-A form; MCS-B: Memory Complaint Scale-B form; SCI: group with subjective cognitive impairment; NSCI: group with no subjective cognitive impairment.

a p-value for likelihood test;

b p-value for Mann Whitney test; Source: Researcher's database.

### MCS-A

According to the intergroup analyses, performed using the Mann-Whitney test, there were no statistically significant differences in the distribution of the MCS-A and other instruments.

In the analyses performed using the likelihood test between the MCS-A sociodemographic variables, total ACE-R and domains, MMSE and SF-8, no significant associations were found (Table 2). However, regarding the CDT, a significant result was observed (p=0.04), indicating the influence of MCS-A results on CDT performance, especially for those who scored >6 for total score (65.8% of those who reported SCI). The univariate logistic regression revealed an OR of 3.83, i.e., individuals reporting MC were about 3.83 times more likely to score >6 on the CDT.

**Table 2:** Results of the associations between instruments used and MCS-A, by group.

		Total			SCI			NSCI		*Odds* *Ratio*	p*
		n	%		n	%		n	%		
MMSE	Altered	17			6	17.5		11	22.4	-	0.591
	Unaltered	66			28	82.3		38	77.5		
ACE-R total	Altered	67			22	64.7		35	71.4	-	0.517
	Unaltered	26			12	35.2		14	28.5		
ACE-R A.O.^a^	Altered	57			22	64.7		35	71.4	-	0.517
	Unaltered	26			12	35.2		14	28.5		
ACE-R Mem.^b^	Altered	40			16	47.0		24	48.9	-	0.863
	Unaltered	43			18	52.9		25	51.0		
ACE-R Fluency	Altered	50			19	55.8		31	63.2	-	0.499
	Unaltered	33			15	44.1		18	36.7		
ACE-R Lang.^c^	Altered	44			15	44.1		29	55.8	-	0.175
	Unaltered	39			19	55.8		20	40.8		
ACE-R V.S.^d^	Altered	51			18	52.9		33	67.3	-	0.185
	Unaltered	32			16	47.0		16	32.6		
CDT	Very poor	22			14	63.6		8	36.3	3.83	0.040
	Poor	17			5	29.4		12	70.5		
	Normal	34			15	34.0		29	65.9		
SF-8	PD^e^	83	100		-	-		-	-	-	0.911
	MD^f^	83	100		-	-		-	-	-	0.394

MCS-A: Memory Complaint Scale-A form; SCI: group with subjective cognitive impairment; NSCI: group with no subjective cognitive impairment;

a ACE-R Attention and Orientation;

b ACE-R Memory;

c ACE-R Language;

d ACE-R Visuospatial;

e Physical Domain;

f Mental Domain;

* Values obtained after analysis using likelihood test.

### MCS-B

The analyses performed with the likelihood test revealed significant associations between the MCS-B and some of the cognitive functions analyzed by the ACE-R, and relevant ORs were obtained with the same variables using univariate logistic regression (Table 3). For the SF-8, no significant association was found for the physical component or mental component.

**Table 3:** Results of associations between instruments used and MCS-B, by group.

		Total			SCI			NSCI		Odds Ratio	p*
		n	%		n	%		n	%		
MMSE	Altered	17	20.4		8	9.6		9	10.8	-	0.200
	Unaltered	66	79.5		20	24.0		46	55.4		
ACE-R total	Altered	57	68.6		25	30.1		32	38.5	5.9	0.002
	Unaltered	26	31.3		3	3.6		23	27.7		
ACE-R A.O.^a^	Altered	57	68.6		21	25.3		36	43.3	-	0.370
	Unaltered	26	31.3		7	8.4		19	22.8		
ACE-R Mem.^b^	Altered	40	48.1		18	21.6		22	26.5	2.7	0.035
	Unaltered	43	51.8		10	12.0		33	39.7		
ACE-R Fluency	Altered	50	60.2		21	25.3		29	34.9	2.6	0.046
	Unaltered	33	39.7		7	8.4		26	31.3		
ACE-R Lang.^c^	Altered	51	61.4		17	20.4		27	32.5	-	0.314
	Unaltered	44	53.0		11	13.2		28	33.7		
ACE-R V.S.^d^	Altered	51	61.4		26	31.3		25	30.1	15.6	<0.001
	Unaltered	32	38.5		2	2.4		30	36.1		
CDT	Very poor	22	26.5		10	12.0		12	14.4	-	0.174
	Poor	17	20.4		3	3.6		14	16.8		
	Normal	44	53.0		15	18.0		29	34.9		
SF-8	PD^e^	83	100		-	-		-	-	-	0.378
	MD^f^	83	100		-	-		-	-	-	0.637

MCS-B: Memory Complaint Scale-B form; SCI: group with subjective cognitive impairment; NSCI: group with no subjective cognitive impairment;

a ACE-R Attention and Orientation;

b ACE-R Memory;

c ACE-R Language;

d ACE-R Visuospatial;

e Physical Domain;

f Mental Domain;

* Values obtained after analysis by likelihood test.

### MCS-A X MCS-B

A column profile test was performed to observe the differences between the two forms of MCS evaluation (Table 4).

**Table 4:** Distinctions between MCS-A and MCS-B, according to column profile test.

		MCS-B	
		NSCI	SCI
**MCS-A**	**NSCI**	34 (61.82%)	15 (53.57%)
	**SCI**	21 (38.18%)	13 (46.43%)

MCS-A: Memory Complaint Scale-A form; MCS-B: Memory Complaint Scale-B form; SCI: group with subjective cognitive impairment; NSCI: group with no subjective cognitive impairment.

There was only 56% concordance between results of the MCS-A and MCS-B (in 47 of the 83 subjects, the presence or absence of SCI matched for the MCS-A and MCS-B).

Among the individuals who did not report SCI according to the MCS-B, 61.82% were also classified as NSCI by the A form. Among those with SCI according to the MCS-B, there was even less agreement, with only 46.43% of individuals also classified as having SCI according to the MCS-A.

Correlation analysis with the two forms of MCS (Table 4) revealed a weak correlation between cognitive performance assessed by the CDT and memory complaint reported by participants, indicating a directly proportional relationship (the more complaints, the less alteration, since the scores are inverted). A weak inversely proportional correlation was observed between memory complaint reported by the informant and visuospatial ability, indicating that the better the performance on this ability the more the informant tended to report the observed complaint. Some tendencies considering the value of p<0.10 can also be noted, where the memory complaint reported by the informant had a moderate inversely proportional correlation with the global evaluation of cognition measured by the ACE-R, corroborating the result related to visuospatial ability. Another relationship between the mental domain of self-perceived health and memory complaint reported by the individual was evident, showing a weak inversely proportional correlation, indicating that the more minor the complaint, the greater the self-perceived health in the mental domain (Table 5).

**Table 5:** Correlation between MCS-A / MCS-B, cognitive performance, and self-perceived health.

	ACE-R						MMSE^f^	CDT^g^	SF-8 (PD)^h^	SF-8 (MD)^i^
	A.O.^a^	Mem^b^	V.F.^c^	Lang^d^	V.S.^e^	Total				
MCS-A	ρ=.198 (0.073)^†^	ρ=.016 (0.887)	ρ=.143 (0.148)	ρ=.153 (0.157)	ρ=.214 (0.052)^†^	ρ=.149 (0.178)	ρ= -.121 (0.275)	ρ=.257 (0.019)*	ρ= -.031 (0.780)	ρ= -.183 (0.097)^†^
MCS-B	ρ= -.163 (0.141)	ρ= -.119 (0.283)	ρ= -.063 (0.571)	ρ= -.175 (0.114)	ρ= -.339 (0.002)*	ρ= -.212 (0.054)^†^	ρ= -.159 (0.150)	ρ= -.072 (0.518)	ρ= -.048 (0.666)	ρ=.069 (0.535)

MCS-A: Memory Complaint Scale-A form; MCS-B: Memory Complaint Scale-B form; ACE-R: Addenbrooke's Cognitive Exam - Revised;

a ACE-R Attention and Orientation;

b ACE-R Memory;

c ACE-R Verbal Fluency;

d ACE-R Language;

e ACE-R Visuospatial;

f Mini-Mental State Examination;

g Clock Drawing Test;

h Physical Domain;

i Mental Domain, p-value presented in parentheses, below correlation coefficient;

* Significant correlations at 5% have p-value < 0.05;

† Significant correlations at 10% have p-value < 0.10.

## DISCUSSION

The objective of the present study was to verify the relationship between memory complaint, cognitive alteration, and self-perceived health in community-dwelling older adults from a population-based study. There was a correlation between SCI and cognitive impairment, especially when considering the perception of the complaint by an informant. No correlations were found between self-perceived health and cognitive impairment.

The CDT was shown to be a sensitive instrument in relation to MC according to the perception of the older adults. A strong association between cognitive deficits related to executive function and MC was also observed in a previous study which used the Prospective Retrospective Memory Questionnaire (PRMQ) to evaluate MC and the CogState to evaluate cognition.^26^ The relationship between memory deficit and decline in executive functions has frequently been reported in the literature, such as impairment associated with Alzheimer's Disease.^27^^,^^28^ The study of Seo et al.^29^ aimed to promote measures sensitive to changes in the cognition of individuals in stages classified as CDR=0.5 compared to older people with CDR=0. One of their main findings was that visual memory and executive function were more compromised in CDR=0.5 individuals.

More notable associations between cognitive impairment and SCI were found with the use of the MCS-B. Global cognition and memory, fluency, and visuospatial domains significantly influenced the presence of SCI. These findings corroborate those of Gifford et al.,^30^ who demonstrated that SCI reported by the informant is related to a greater global cognitive decline, since the trajectory of cognitive decline tends to be worse when compared to SCI reported only by the participant or only by the informant. These results contrast with the findings of Thompson et al.^31^ in the SCI evaluation reported by the subject and informant when comparing groups of cognitively healthy elderly, older adults with MCI, and older adults with dementia. SCI was evaluated using the PRMQ and cognition using the MMSE, and as a main result the authors found that there was a significant correlation between the prospective and retrospective memory reported by the older adults and the informant only in the group with dementia. In addition, the prospective SCI of the older adults did not correlate with the MMSE scores, while the SCI reported by the informant correlated with the MMSE only in the dementia group.

Regular and poor self-perceived health has been studied as an important factor for the prediction of dementia, cognitive impairment, and SCI.^32^^-^34 Individuals with better self-perceived health tend to report less SCI,^33^ but the correlation between self-perceived health and SCI failed to confirm this result. In the present study, the results indicated that the sample, in general, has a self-perceived health that can be considered as regular, since the means of the SF-8 domain scores are close to the mean value (50 points).^35^ This was the first Brazilian study to evaluate self-perceived health using the SF-8, an instrument for which more evidence is required regarding its specificity and sensitivity for screening in the Brazilian population.

Regarding the MCS-A and MCS-B instrument forms, both are designed to measure SCI in the same individual. Thus, it was expected that, overall, results would exhibit agreement; however there were considerable differences between values obtained on the MCS-A and MCS-B. Some studies have found the informant´s perception of the elderly subject with SCI to be a more reliable predictor of cognitive impairment than the elderly subject´s report.^11^^,^^36^^-^38 The SCI, when not associated with objective memory alteration, may reflect a distorted awareness of the current cognitive state compared to the past. While anosognosic individuals regard their cognitive abnormality as normal, individuals with SCI may consider their normal cognitive state to be abnormal. Moreover, SCI can be considered a possible consciousness deficiency.^39^ Thus, there is a need to include and value the perception of someone who knows the older individual sufficiently well to evaluate SCI globally, to ensure that cases of elderly with cognitive impairment are evaluated by health professionals.

The present study has some limitations regarding the number of individuals evaluated and the choice of a little-used screening instrument for one of its main measures. In addition, objective cognitive evaluation of the informants, who were often not only elderly themselves, but also companions of the study subjects, was not performed. It should be noted that these findings cannot be extrapolated to the general population. One of the highlights of this study was the value given to the informant's report for the SCI study, which proved to be fundamental in relation to the cognitive impairment.

Due to the importance of identifying the pre-clinical stages of dementia, SCI and its related factors are gaining greater visibility in the current scientific scenario, in a bid to establish early and preventive treatment. In the present study, SCI reported by the older adults was associated only with executive function. However, when the informant reported SCI, there was a significant association with overall cognitive performance and other specific cognitive domains.

These results highlight the need for evidence regarding the etiology of SCI and the need for further assessment of the mental health of the community-dwelling older adults in an effort to promote changes in existing public policies, principally the crucial role of the informant in this evaluation. Many factors can influence an individual´s perception of their memory. Given that the aging process is heterogeneous, other factors can be investigated as predictors of SCI, such as social and family support and advanced activities of daily living.
